# OUTBREAK OF TINEA GLADIATORUM IN WRESTLERS IN TEHRAN (IRAN)

**DOI:** 10.4103/0019-5154.43219

**Published:** 2008

**Authors:** Shahindokht Bassiri-Jahromi, Ali Asghar Khaksar

**Affiliations:** *From Department of Medical Mycology, Pasteur Institute of Iran, Tehran, Iran*

**Keywords:** *Dermatophytosis*, *infection control*, *Iran*, *tinea corporis*, *tinea gladiatorum*, *trichophyton tonsurans*, *wrestling*

## Abstract

**Background:**

In recent years, skin diseases in wrestling have finally received the attention they deserve. Outbreaks of tinea corporis are often associated with sports involving extensive bodily contact; such sports include wrestling. Tinea corporis gladiatorum is primarily caused by *Trichophyton tonsurans*, infecting wrestlers at alarming rates. The management of skin infections in wrestlers and other athletes in sports involving skin-to-skin contact entails numerous challenges, from making an accurate diagnosis to determining eligibility for playing the sports. To control outbreaks, we conducted an epidemiologic investigation. The purpose of this article is to determine the prevalence of tinea corporis gladiatorum in wrestlers in Tehran, Iran.

**Materials and Methods:**

A study of dermatophytosis was carried out during the period of March 2004 to December 2005 on 612 mycological proven cases of dermatophytosis found in male wrestlers in Tehran. Mycological examination consisted of culturing of pathologic material followed by direct microscopic observation. Diagnosis was based on macroscopic and microscopic characteristics of the colonies.

**Results:**

*T. tonsurans* was the predominant dermatophyte, accounting for >90% of all tinea corporis gladiatorum isolates during the 2 year analysis. Tinea corporis gladiatorum was found to be more frequent in individuals between the ages of 10 and 20 years of age (72.7%). Wrestlers with tinea corporis gladiatorum were predominantly from wrestling clubs in southern and southeastern Tehran. Transmission of tinea corporis is primarily through skin-to-skin contact.

**Conclusion:**

Rapid identification and treatment of tinea corporis gladiatorum is required to minimize the disruption of team practices and competitions. Infection with dermatophytes can disqualify a wrestler from competing in matches, and thus, vigilant surveillance and rapid initiation of treatment is important to prevent the suspension of team practices and competitions.

## Introduction

One of the most common types of infections that can occur in wrestlers is a fungal infection known as tinea corporis gladiatorum. Characterized by well-defined, red, scaling plaques located on the head, neck and upper extremities, tinea corporis gladiatorum epidemics have been reported in numerous wrestling teams ranging in prevalence from 24% to 77%.^1^

Tinea corporis gladiatorum is extremely common in wrestling and other sports involving extensive skin-to-skin contact and can result in outbreaks. Wrestlers acquire tinea corporis from direct skin-to-skin contact; thus, the predilection for the head, neck and upper extremities. Wrestling as a part of the National Collegiate Athletic Association (NCAA) ranks number one in the frequency of cutaneous infections.^2^

Lesions often occur on the arms, torso, head and neck, corresponding to the areas of greatest contact between combatants. Wrestling requires close body contact and often results in skin abrasions that are a perfect opportunity for person-to-person transmission. However, dermatophytes have been isolated from several inanimate objects, including hairbrushes, combs, pillowcases, other bedding material and dormitory floors.^3^ Inanimate objects or fomites may be responsible for prolonged transmission of ringworm infections;^4^ the competitive wrestling environment includes many omits as possible source of contagion. Tinea corporis gladiatorum is caused by dermatophytes, usually of the genus *Trichophyton*, affecting both humans and animals. The fungus causes a characteristic lesion which is often clear in the center with a rough, scaly circular border. Lesions vary in size from very small circular patches to large patches.^5^

The primary mode of transmission in wrestlers is to person-to-person contact. Clinical features of tinea gladiatorum may or may not be consistent with those found in the general population.^6^

The aim of this study is to determine the prevalence of tinea corporis gladiatorum and identify the primary causative agents of dermatophytosis and other related factors in wrestlers in Tehran, Iran.

## Materials and Methods

This study was conducted from March 2004 to December 2005. We have published a leaflet showing the clinical characteristics and methods of diagnosis of tinea gladiatorum. These leaflets were distributed to the wrestler clubs all over Tehran and around Tehran via the wrestler federation, asking the members to participate in the survey. Directing managers or coaches of the wrestler clubs who agreed to participate in the survey were asked to complete application forms describing the location of the club and the number of club members who agreed to participate in the survey. The clinical examination of self-referred athletes was performed. Wrestlers who showed suspected lesions were referred to Medical Mycology Pasteur Institute of Iran.

Demographic information requested in the questionnaire included age, number of family, members and residence conditions and history of cutaneous infection in the family members. The participants were also monitored for current or previous possible tinea gladiatorum eruptions.

The clinical diagnosis and detailed history of each wrestler were recorded. Specimens from suspected lesions were collected in sterile Petri dishes. All collected specimens were submerged in potassium hydroxide solution in order to examine characteristic fungal elements. The specimens were cultured on sabouraud glucose agar (oxoid, Basingstoke, United Kingdom) supplemented with 0.05 mg/l chloramphenicol and 0.5 mg/l cyclohexamide. Cultures were incubated for 4 weeks at 25°C. The identification of dermatophytes was performed on the basis of both macroscopic and microscopic appearance. Slide cultures and other confirmatory tests such as Trichophyton agar slants, urea agar slant, potato glucose agar, cornmeal, rice grain medium and an *in vitro* hair perforation test were performed when necessary.^7^–^8^

## Results

A total of 893 male wrestlers, aged 6-42 years, from 173 active clubs in Tehran were examined, most of whom were members of wrestling clubs in southern or southeastern Tehran. The mean age of the wrestling team was 18.2 years. Tinea corporis in 64 cases was documented among the family members of infected wrestlers. Most of the patients came from wrestling clubs in southern and southeastern Tehran.

The most frequently isolated etiological agent was *Trichophyton tonsurans* (92.6%), followed by *Trichophyton rubrum* (2.8%), *Trichophyton mentagrophytes* (1.75%), *Epidermophyton floccosum* (1.75%), *Trichophyton violaceum* (0.43%) and *Trichophyton verrucosum* (0.43%). *Microsporum canis* (0.2%) infections occurred sporadically among patients.

*Tinea corporis gladiatorum* was found to be more prevalent between the ages of 10 to 30 years. In 64 cases (10.8%), tinea corporis was present among the family members of infected wrestlers.

## Discussion

Contact sports provide an excellent setting for the transmission of communicable disease. Outbreaks of fungal infections are common in contact sports such as wrestling, judo and kung fu because of the close physical contact and trauma to the skin involved in these sports. Several outbreaks of tinea corporis or ringworm have recently been reported in high school and college wrestling.^9^–^17^

Tinea gladiatorum outbreaks have been caused by *T. tonsurans*. The primary mode of transmission in wrestlers is to the skin-to-skin contact.^18^

Dermatophytosis is extremely common in wrestling and other sports with extensive skin-to-skin contact. Asymptomatic carriers may be an important source of fungal organisms.^18^,^19^ Risk factors for becoming a carrier include active infection during the sporting season, history of head and neck tinea infection, failure to wear headgear and failure to wash practice clothes at least once a week.^20^ By promptly identifying the infected athletes and excluding them from direct contact with other wrestlers can help to reduce tinea occurrences.^21^

To our knowledge, this is the largest reported series of patients with tinea gladiatorum and the only one to describe such infections in Iran. There are more than 90 wrestling clubs and 25,000-30,000 wrestlers in Tehran. Wrestling is a national sport in Iran and plays an important role in Iranian entertainment. Subsequently, the practicing of wrestlers can lead to direct and indirect exposures to and transmission of dermatophytes between wrestlers along with positive observers.

*T. tonsurans* is an anthropophilic dermatophyte and was the main cause of tinea corporis in wrestlers in this study. The incidence of *T. tonsurans* infection is dynamically changing in various parts of the world.^22^ Although *T. tonsurans* rarely occurred in Iran,^23^ in 1995, it began to spread sporadically in Tehran and since then it has dramatically increased throughout Iran. This study demonstrates a high occurrence of *T. tonsurans* infection in wrestlers (92.6%). In recent years it has become the predominant cause of tinea corporis in adolescents.^24^–^25^ *T. tonsurans* is one of the main causes of dermatophytosis in Iranian wrestlers. Skin infections due to *T. tonsurans* have become a significant health problem affecting children, adolescents and sometimes adults. Therefore, correct diagnosis and treatment of the active disease is important. Infection can also be reduced by screening of possible carriers and treatment of asymptomatic carriers and their environment.^3^ *T. tonsurans* remains the predominant causative agent in North America and currently accounts for >95% of tinea capitis in the United States.^18^–^19^

Since the 1950s, *T. tonsurans* has spread to North America.^19^ A rapid increase in the incidence of *T. tonsurans* resulting in tinea capitis has been noted in Canada. Canadian studies reported an incidence of 9% in 1985 and 76% in 1996.^26^ In Europe, an increasing number of *T. tonsurans* infections in recent years suggest its return to this area. In 1999, Fitowski and Ratka^27^ reported an epidemic outbreak of *T. tonsurans* in 23 village children in Poland. In 1995, an outbreak of tinea corporis occurred in members of a wrestling team in Sweden. Wrestlers from a USA team visiting Sweden were the suspected source of this epidemic.^28^ Recently, *T. tonsurans* was also reported to occur in the United Kingdom and was found to be the predominant cause of tinea capitis, accounting for 72% of the observed infections in Birmingham.^29^ Other outbreaks of *T. tonsurans* were also reported in southeastern London (United Kingdom)^30^ and among school children in Spain.^31^

This particular study focuses on its occurrence in wrestlers in Tehran during recent years. In this study, incidence of tinea corporis gladiatorum occurred in males between the ages of 10and 20 years. Occlusion has been postulated to increase hydration of the underlying skin and emission of carbon dioxide from the skin, which could favor dermatophyte growth.^32^,^33^ Tinea corporis in 64 cases (10.8%) was documented among the family members of infected wrestlers. Most of our patients came from wrestling clubs in southern and southeastern Tehran. This is the first and largest outbreak of tinea corporis gladiatorum to be reported in Tehran. Appropriate control measures have not yet been established.

Tinea corporis is spread through direct contact with infected individuals and may also occur due to contact with infectious spores on inanimate objects such as clothing, mats, etc. However, the presence of a dermatophyte on a fomite or as part of a carrier sate does not affirm it as the definitive source. The principles of an infectious disease require a viable organism, a susceptible host and an appropriate environment for clinical infection to occur. Several authors suggest that some wrestlers may be asymptomatic carriers and act as reservoirs.^6^–^34^–^35^ Tinea gladiatorum is highly contagious and is a result of the presence of *T. tonsurans*. As infection with dermatophytes can disqualify a wrestler from competing in matches, vigilant surveillance, prevention, rapid identification; further, treatment of tinea gladiatorum is vital to reduce the suspension of team practice and competition. Appropriate treatment can mitigate an infection and potentially prevent recurrence. In addition, physicians must know when to disqualify a wrestler and how to prevent an outbreak through appropriate hygienic and immediate diagnostic measures.^36^,^37^ In recent decades, the improvement of hygiene standards and earlier treatment may have decreased widespread infections.

We need to study all aspects of this infection in this population in order to develop strategies to deal with it. Because infection with dermatophytes can disqualify a wrestler from competing in matches, vigilant surveillance, prevention, rapid identification and treatment of tinea gladiatorum is vital to reduce the suspension of a team's practice and competition.

Good personal hygiene helps prevent the spread of ringworm. Showering thoroughly after practices and competitions and washing uniforms after they are used with antibacterial detergent is an effective means in achieving appropriate hygiene.^6^,^35^ Furthermore, uniforms and practice clothing should not be shared among wrestlers.^35^

Tinea corporis gladiatorum can be found quite frequently among high school wrestlers. Without a thorough knowledge of tinea gladiatorum, wrestling is compromised as a sport. Vigilant surveillance and rapid initiation of therapy is important to prevent suspension of team practices and competitions due to infection with dermatophytes. Awareness of these infections may facilitate the implementation of early treatment and preventive measures.^35^

We suggest focusing our efforts on studying the person-to-person transmission, studying when return to competition techniques such as the use of skin barriers^34^ and pharmacologic prophylaxis.^37^,^38^ We would suggest the continuation of common-sense hygiene measure, including showering after very encounter, washing practice clothes daily and disinfecting mats daily.^36^ Until we have more definitive answers about ringworm in wrestlers, it is impossible to have sufficient infection control and prevention plans.

Attention should be focused on primary and secondary prevention as well as treatment. Educating wrestlers, coaches, parents and members of the medical community about skin infections and their prevention, recognition and treatment is crucial and a part of our continuing effort. The prevention of tinea should be a major priority in wrestling. Prevention begins with cleaning all the mats before and after practices with a hospital grade disinfectant. Secondly, wrestlers should be educated on symptoms and trained to inspect their own bodies daily. Thirdly, wrestlers must wash all workout equipment daily and be sure to wash knee pads and headgear twice a week. Fourth, wrestlers should shower and use an antibacterial soap and selenium shampoo immediately after workouts. It is also important to avoid drying of skin as it is more susceptible to infection. Finally, when a lesion is noticed, the individual must consult their physician or allelic trainer and use the appropriate medication. The lesion should be covered prior to wrestling according to NCAA guidelines outlined below.^39^

Hygienic measures, such as mandatory showers before and after practice, use of antibacterial soaps and daily washing of practice gear, may be the most effective means in preventing skin infections.

This study highlights a common problem in many areas of the world and suggests that further measures regarding public health and personal hygiene must be undertaken in order to reduce the risk of tinea gladiatorum.^5^,^40^ In particular, greater and more efficient sanitary control should be implemented in communal environments such as gymnasia, farms, factories, swimming pools, changing rooms of sports clubs and public showers.

More research concerning different treatment regimens in the wrestling environment is needed to define the optimal treatment to quickly return wrestlers to competitions without placing other wrestlers at risk of infection. Intuitive hygiene practices have been suggested to prevent the spread of infection, but have not yet been substantiated.^36^

Knowledge of the most common agents producing infectious disease outbreaks in specific sports can be used to guide targeted prevention efforts. Surveillance of the frequency of infections per team each season will also allow athletic staff to identify outbreaks.^1^

In conclusion, these data reiterate the continued predominance of *T. tonsurans* as the principal pathogens in cutaneous fungal infections in Tehran. *T. tonsurans* remain the most prevalent pathogen as tinea gladiatorum in wrestlers. The most prevalent fungal pathogen was *T. tonsurans* (92.6%), followed by *T. rubrum, T. mentagrophytes, E. floccosum, T. violaceum* and *T. verrucosum* and *M. canis.*
